# Assessment of socio demographic factors on termination of pregnancy in Ghana

**DOI:** 10.1002/nop2.2152

**Published:** 2024-04-05

**Authors:** Mavis Akosuah Serwah Armah‐Mensah, Isaac Armah‐Mensah

**Affiliations:** ^1^ Department of Health Policy, School of Public Health University of Ghana Accra Ghana; ^2^ Department of Computer Science University of Cape Coast Cape Coast Ghana

**Keywords:** Ghana, maternal mortality, nurse, nursing, socio demographic factors, termination of pregnancy

## Abstract

**Aim:**

Assessing the socio‐demographic factors on termination of pregnancy in Ghana.

**Design:**

Cross‐sectional study, using data source from the Demographic Health Survey (DHS).

**Methods:**

Data pooled from the most recent DHS conducted in Ghana, with variables of interest with rural and urban population coverage. A systematic search of the literature was performed using PubMed, Google Scholar and Elsevier PubMed for the secondary data. Descriptive and logistic regression analysis was performed using Python Pandas' software to estimate the independent effects of the socio‐demographic factors on termination of pregnancy in Ghana.

**Results:**

Reported using odds and adjusted OR AOR at 95% confidence level and statistical significance at a *p*‐value of (*p* > 0.05). Age, place of residence, occupation, currently pregnant, woman's individual sample weight, completeness of current pregnancy, living children + current pregnancy, ethnicity and number of living children significantly predicted the outcome variable.

**Patient or Public Contribution:**

Nurses have an important role to play in providing support, education and counselling to people, and must be equipped with the knowledge and skills (including non‐judgmental and compassionate care) necessary to provide care that is sensitive to the diverse needs of people from different socio‐demographic backgrounds.

## 1 INTRODUCTION

Maternal healthcare, especially among women of reproductive age, is an important part of the public health system (Geetika et al., 2015). Quality of life in pregnant women is an important issue both for women's and foetus' health. There is a prevailing high fertility rate for women in Sub‐Saharan Africa (SSA). UN estimates the total fertility rate of‐SSA at 4.7 births per woman in 2015–2020, more than twice the level of any other world region (United Nations, 2019). One of the major reasons for the high fertility rates in most countries in SSA, including Ghana is that a considerable number of its women of reproductive age are not contraceptive users (Ahinkorah et al., 2020). However, many of the reproductive‐aged women are at risk of unwanted pregnancies and, therefore, may seek ways to terminate them (Bongaarts, 1997). Between the years 2010 and 2014, about 55.7 million terminations of pregnancies in the form of abortions occurred annually worldwide, where 88% of them were from developing countries (Ganatra et al., 2017). A newly released model of estimates for pregnancies and abortions in the world shows that some 266,000 abortions were done in Ghana within four years from 2015 to 2019 (Ghana Business News, 2022).

## 2 BACKGROUND

Maternal mortality is the second most common cause of death among women in Ghana, and more than one in 10 maternal deaths (11%) are the result of unsafe induced abortions (Ghana Statistical Service, Ghana Health Service, & Macro International, 2007). Pregnancy termination can happen in the form of abortion, miscarriage, menstrual regulation and stillbirth. The rate at which pregnancies are terminated has remained high in middle and lower‐income countries (MLIC'S) around the world, and this reflects a trend of mistimed, unwanted and unintended conceptions (World Health Organization, 2016). According to Haddad and Nour (2009), of the millions of women who terminate an unintended pregnancy each year, approximately half are recipients of an ‘unsafe’ intervention; unsafe abortions are performed by unskilled individuals in environments that do not meet medical standards. Religion and spirituality also play a very important role in the youths' attitudes toward termination of pregnancy. According to Selebalo‐Bereng and Patel (2019), Muslims reported greater disapproval than other religious groups. They also found out that Christian youth had the most negative attitudes to pregnancy termination for ‘Traumatic reasons’ and ‘When women's health/life’ was threatened. This invariably generates a lot of concerns for maternal healthcare, especially among women of reproductive age in the MLIC's.

In Ghana, termination of pregnancy remains a major public health challenge despite the apparent liberalization of the law on abortion over two decades. Although termination of pregnancy in Ghana is legally permissible, it can only be conducted at a government hospital; registered private hospital, clinics registered under the Private Hospitals and Maternity Homes Act, 1958 (No. 8) and a place approved by the Minister of Health by a Legislative Instrument. The contention is that the current law on abortion makes enforceability difficult and leaves room for untrained personnel to engage in dangerous abortion procedures and that there is a need for law reform (Morhee & Morhee, 2006).

In Ghana, termination of pregnancy has gained appreciable attention in reproductive health discussions (Lithur, 2004). This is surprising given the country's restrictive laws, which permit abortion only in instances where conception is the result of defilement, rape, or incest or if pregnancy poses a threat to the health of the woman (Lithur, 2004).

A couple of studies have identified socio‐demographic factors that are associated with pregnancy termination in developing countries, and these include age of the women, marital status, educational level, employment status and total number of children. These factors have significantly been associated with an increased risk of pregnancy termination (Adjei et al., 2015; Arambepola et al., 2016; Dankwah et al., 2018; Maina et al., 2015). According to Dankwah et al. (2018), women who are employed, cohabit with a partner, have intermediate levels of education, and are considered middle‐class or wealthy are more likely than their counterparts to report having terminated a pregnancy. Klutsey and Ankomah (2014), indicated that marital status, employment status, number of total pregnancies, and knowledge about contraception were found to be associated with induced abortion. They did not, however, find any relationship between pregnancy termination and maternal age, education, contraceptive use, or religion. women who are capable of taking reproductive health decisions, age, level of education, contraceptive use and intention, place of residence and parity are associated with pregnancy termination (Seidu et al., 2020). According to Ahinkorah et al. (2021), the socio‐demographic determinants of pregnancy termination among AGYW are age, level of education, marital status, exposure to radio and television, parity and community literacy level. The findings provide the needed information for designing health interventions to reduce unwanted pregnancies and unsafe termination of pregnancies in countries with high‐fertility rates in SSA.

Termination of pregnancy exhibits diverse forms of risk that include secondary infertility, ectopic pregnancy and foetal loss increase (Hogue, 2008). Studies have consistently shown that induced abortion increases the incidence of subsequent preterm delivery and depression (Thorp et al., 2003), which can ultimately affect a woman's decision to have more children in the future. Pregnancy termination has therefore been found as one of the key issues that need to be addressed to attain Sustainable Development Goal 3 by 2030 (Manandhar et al., 2018).

## 3 THE STUDY

### 3.1 Design

A cross‐sectional study was used in collecting data at a single point in time from a sample of individuals representing the population of interest.

### 3.2 Method

Existing data sources, the DHS national survey, were used to identify a sample of women who have undergone termination of pregnancy to collect data on their socio‐demographic characteristics, such as age, ethnicity, education level, income, marital status and residence.

Data was pooled from the most recent DHS conducted in Ghana, including all the variables of interest which had the coverage for both rural and urban populations. Permission to utilize these data sets was granted after registering with the DHS programme manager. A systematic search of the available literature was performed in September to December 2022 using the following databases: PubMed, Google Scholar and Elsevier for the secondary data. The search strategy was to determine search concepts and types of studies on the topic. The keywords (and their combinations) adopted for the manuscript are the following: socio‐demographic factors and termination of pregnancy. Separate searches for each primary database combined subject headings, terms and key text words with the Boolean operators (AND) and (OR), accordingly. All eligible pregnant women between the ages of 15 and 49 selected for the individual were used for the study. The socio‐demographic variables included age, residence, currently working, religion, highest educational level for both woman and husband, wealth index, husband partner occupation, religion and ethnicity as independent variables or determinants used as predictor variables.

### 3.3 Analysis

The socio‐demographic factors among pregnant women of reproductive age 15–49 years were examined and analysed. Socio‐demographic factors associated with termination of pregnancy were identified by estimating binary and multivariable logistic regression models. Non‐pregnancy termination was used as the reference group or baseline for the analysis.

Data was analysed using Python Pandas. Weights were applied to all the descriptive statistics using women's individual sample weight (v005) for the individual recode model. Descriptive statistics were used to summarize the proportion for categorical variables, unadjusted and AORS with 95% confidence interval (CI), estimated in a logistic regression to identify the determinants of termination of pregnancy and a number of explanatory variables at a *p* value of <0.05 considered statistically significant to assess and strengthen the association. The final selection for inclusion criteria in the final logistic model was based on a significant association of sociodemographic factors and effects on termination of pregnancy.

The datasets were carefully checked for missing values, which were excluded and weighted with the appropriate sampling weights as per the DHS sampling scheme. Three levels of analyses (univariate, bivariate and multivariate) were employed. At the univariate level, descriptive statistics related to the characteristics of the study population and socio‐demographic factors were generated through frequency and percentage. Using the Pearson chi‐square test, unadjusted binary logistic regression analyses were adopted at the bivariate level to investigate the independent effect of each explanatory variable on the outcome variable. Chi‐square tests were performed to assess differences in categorical variables. Two models of multivariable logistic regression were performed to assess the relationship between socio‐demographic and other variables on termination of pregnancy. Women were asked whether they had ever experienced termination during pregnancy, and if so, what led to it. Additionally, women were asked whether they had ever had a pregnancy loss as a consequence of their pregnancy at the multivariate level. Binary logistic regression models were used to measure the odds ratio (OR) of the association between socio‐demographic factors as the explanatory variables on the outcome and termination of pregnancy. The unadjusted and adjusted binary logistic regression analyses of the association between socio‐demographic and termination of pregnancy were carried out at the multivariate level. Possible associations between each of the explanatory variables and the outcome variable were assessed by observing the *p*‐values. The results of the explanatory variables were expressed as OR with 95% confidence intervals.

## 4 Ethics

Since it was a study using secondary data ethical approval was not required.

## 5 RESULTS

5 ethnic groups in Ghana on termination of pregnancy are presented in Table 1.

**TABLE 1:** Ethnic groups in Ghana and termination of pregnancy.

Ethnic group	Weighted sample	Weighted percentage (%)
Akan	4132	44.0
Mole Dagbani	3453	36.7
Ewe	1118	11.9
Ga Dangme	519	5.5
Other ethnic groups	174	1.9

### 5.1 Descriptive statistics

Table 1 describes the ethnic groups in detail with their frequencies and percentages of pregnancy termination. The prevalence of pregnancy termination in each of the ethnic groups in terms of weighted percentage ranged from 1.9% (other ethnic groups) to 44.0% (Akan).

The result is based on a weighted sample of 9396 women of reproductive age (15–49) years whose characteristics are summarize in Table 2. Majority of the women, 1759 (18.7%), were within the ages of 15–19 years, 4794 (51.0%) were living in the rural area, 7170 (76.3%) were Christians, 4854 (51.7%) had secondary education with husband or partners who had no education 4888 (52.0%) and aged above 55 years 4455 (47.4%).

**TABLE 2:** Socio‐demographic characteristics of women of reproductive age.

Characteristic	Frequency	Percentage
Age in groups		
15–19	1756	18.7
20–24	1571	16.7
25–29	1564	16.6
30–34	1343	14.3
35–39	1260	13.4
40–44	1032	11.0
45–49	870	9.3
Husband/partner age		
55 and above	4455	47.4
35–44 years	1884	20.1
25–34 years	1651	17.6
45–54 years	1141	12.1
15–24 years	265	2.8
Highest educational level		
No education	2281	24.3
Primary education	1747	18.6
Secondary education	4854	51.7
Higher/tertiary education	514	5.5
Husband/partner education		
No education	4888	52.0
Secondary	3169	33.7
Higher/tertiary	696	7.4
Primary	643	6.8
Type of place of residence		
Urban	4602	49.0
Rural	4794	51.0
Husband/partner occupation		
Working	6299	67.0
Not working	3097	33.0
Currently pregnant		
No	8717	92.8
Yes	679	7.2
Duration of current pregnancy		
First trimester	8921	94.9
Second trimester	255	2.7
Third trimester	220	2.3
Wealth index		
Poorest	2335	24.9
Poor	1759	18.7
Middle	1902	20.2
Richer	1771	18.8
Richest	1629	17.3
Current marital status		
Married	4243	45.2
Never in union	3041	32.4
Living with a partner	1213	12.9
No longer living together/separated	370	3.9
Widow	269	2.9
Divorce	260	2.8
Religion		
Christianity	7170	76.3
Islam	1726	18.4
Others	274	2.9
Traditionalist	226	2.4
Completeness of current pregnancy		
Uncompletedness of current pregnancy information	8717	92.8
Completeness of current pregnancy information	679	7.2
Respondent currently working		
No	2635	28.0
Yes	6761	72.0
Living children + current pregnancy		
No living children current pregnancy	2805	29.9
5 or more living children + current pregnancy	1653	17.6
2 living children + current pregnancy	1391	14.8
3 living children current pregnancy	1173	12.5
4 living children current pregnancy	1011	10.8
1 living child + current pregnancy	1363	14.5
Number of living children		
0 living children	2948	31.4
1 living child	1380	14.7
2 living children	1367	14.5
3 living children	1134	12.1
4 living children	988	10.5
5 or more living children	1579	16.8

Majority of the women, 7020 (74.7%), were working with 6299 (67.0%) of husband or partners working. 8717 (92.8%) of the women were currently not pregnant, 2335 were (24.9) poorest, 4243 (45.2) were married, with 2948 (31.4%) having no living child.

Most of the women 8921 (94.9%), were pregnant in their first trimester, currently working 6761 (72.0%), Uncompletedness of current pregnancy information 8717 (92.8%), no living children + current pregnancy 2805 (29.9%).

The results of Table 3 are from a logistic regression analysis where the outcome variable is “Ever had terminated pregnancy.” The model converged successfully with a final function value of 0.47 and 6 iterations. There are a total of 9396 observations in the dataset. The Pseudo *R* ^2^ value is 0.09, indicating that the model explains about 9.3% of the variation in the outcome variable. Reporting on the OR of the binary logistic regression.

**TABLE 3:** Binary and multivariable logistic regression model.

	Binary logistic regression						Multivariable logistic regression					
	OR	SE	*z*	*p* > |*z*|	[0.025	0.975]	AOR	SE	*z*	*p* > |*z*|	[0.025	0.975]
Household no.	1.00	0.00	−0.50	0.62	−0.01	0.00	1.00	0.00	−0.50	0.62	−0.01	0.00
Age in 5 years group	1.32	0.03	11.21	0.00	0.23	0.33	1.32	0.03	11.21	0.00	0.23	0.33
Highest educational level	1.06	0.03	1.91	0.06	0.00	0.12	1.06	0.03	1.91	0.06	0.00	0.12
Husband partner educational level	1.04	0.03	1.30	0.20	−0.02	0.09	1.04	0.03	1.30	0.20	−0.02	0.09
Type of place of residence	1.31	0.06	4.39	0.00	0.15	0.39	1.31	0.06	4.39	0.00	0.15	0.39
Respondent occupation	1.70	0.16	3.22	0.00	0.21	0.85	1.70	0.16	3.22	0.00	0.21	0.85
Husband partner occupation	1.40	0.13	2.66	0.01	0.09	0.59	1.40	0.13	2.66	0.01	0.09	0.59
Currently pregnant	0.15	0.25	−7.63	0.00	−2.42	−1.43	0.15	0.25	−7.63	0.00	−2.42	−1.43
Woman's individual sample weight	1.00	0.00	5.98	0.00	0.00	0.00	1.00	0.00	5.98	0.00	0.00	0.00
Duration of current pregnancy	0.87	0.12	−1.13	0.26	−0.37	0.10	0.87	0.12	−1.13	0.26	−0.37	0.10
Wealth index	0.98	0.02	−0.79	0.43	−0.06	0.03	0.98	0.02	−0.79	0.43	−0.06	0.03
Current marital status	0.99	0.03	−0.34	0.74	−0.07	0.05	0.99	0.03	−0.34	0.74	−0.07	0.05
Religion	1.04	0.05	0.85	0.40	−0.05	0.13	1.04	0.05	0.85	0.40	−0.05	0.13
Completeness of current pregnancy information	0.13	0.21	−9.78	0.00	−2.48	−1.65	0.13	0.21	−9.78	0.00	−2.48	−1.65
Living children + current pregnancy	0.92	0.02	−4.51	0.00	−0.12	−0.05	0.92	0.02	−4.51	0.00	−0.12	−0.05
Primary sampling unit	1.00	0.00	−2.22	0.03	0.00	0.00	1.00	0.00	−2.22	0.03	0.00	0.00
Ethnicity	0.89	0.03	−4.49	0.00	−0.16	−0.06	0.89	0.03	−4.49	0.00	−0.16	−0.06
Respondent currently working	0.99	0.15	−0.05	0.96	−0.31	0.29	0.99	0.15	−0.05	0.96	−0.31	0.29
Husband partner age	0.89	0.04	−3.37	0.00	−0.19	−0.05	0.89	0.04	−3.37	0.00	−0.19	−0.05
Number of living children	0.89	0.02	−4.99	0.00	−0.17	−0.07	0.89	0.02	−4.99	0.00	−0.17	−0.07
Sample strata for sampling errors	0.99	0.01	−2.48	0.01	−0.03	0.00	0.99	0.01	−2.48	0.01	−0.03	0.00

The coefficients (coef) and standard errors (SE) of the independent variables are also provided. The *p*‐value (*p* > |*z*|) is used to determine the significance of the variable in the model. A *p*‐value of <0.05 is considered significant. From the results, it appears that age in the 5‐year group, type of place of residence, respondent occupation, currently pregnant, woman's individual sample weight, completeness of current pregnancy, living children + current pregnancy, ethnicity and number of living children are significant predictors of the outcome variable. The other variables have *p*‐values >0.05, indicating that they are not significant predictors of the outcome variable.

Socio‐demographic variables predicting termination of pregnancy in Ghana, that have the highest coefficients and the lowest *p*‐values (indicating that they are statistically significant predictors) are the 25–29 years, (AOR = 6.2, 95% CI: 1.8–2.5) 30–34 years, (AOR = 8.7, 95% CI: 2.1–2.9) 35–39 years, (AOR = 9.9, 95% CI: 3.1–14.6) and 45–49 (AOR = 10.3, 95% CI: 2.2–3.1) years women in the groups. These groups have OR and AOR >8, indicating that they are strong predictors of the outcome variable, as compared to the 15–19 year group as the reference.

With higher education, and termination of pregnancy from the Table 4 provided, the highest educational level that has the strongest negative coefficient and the lowest *p*‐value (indicating that it is a statistically significant predictor) is “Higher/Tertiary” level of education, with an OR of 0.66 and an AOR of 0.49, indicating that individuals with this level of education are less likely to have a certain outcome compared to the reference group of “No Education”. Suggesting that having a higher level of education is negatively associated with the outcome variable, but other groups, “Primary” and “Secondary”, have OR >1 and adjusted ratios <1, indicating that these groups are positively associated with the outcome variable with no education as the reference.

**TABLE 4:** Multivariable logistic regression indicating levels predicting outcome variables highly.

	Coef.	SE	*z*	*p* > |*z*|	0.025	0.975	OR	AOR
Intercept	−5.0	0.2	−24.6	0.0	−5.4	−4.6	0.0	0.0
Age in 5 years group								
15–29 years	Base							
20–24 years	1.75	0.17	10.20	0.00	1.42	2.09	5.78	4.13
25–29 years	2.18	0.18	11.99	0.00	1.83	2.54	8.89	6.22
30–34 years	2.56	0.19	13.39	0.00	2.18	2.93	12.90	8.87
35–39 years	2.69	0.20	13.52	0.00	2.30	3.08	14.67	9.94
40–44 years	2.52	0.21	12.03	0.00	2.11	2.93	12.39	8.22
45–49 years	2.76	0.21	12.90	0.00	2.34	3.17	15.72	10.35
Highest educational level								
No education	Base							
Tertiary	−0.41	0.15	−2.75	0.01	−0.71	−0.12	0.66	0.49
Primary	0.16	0.09	1.82	0.07	−0.01	0.34	1.18	0.99
Secondary	0.02	0.09	0.23	0.82	−0.15	0.19	1.02	0.86
Husband partner educational level								
No education	Base							
Higher/tertiary	0.06	0.12	0.50	0.61	−0.18	0.31	1.06	0.83
Primary	0.12	0.12	0.99	0.32	−0.11	0.35	1.12	0.89
Secondary	0.07	0.09	0.78	0.44	−0.11	0.25	1.07	0.90
Type of place of residence								
Rural	Base							
Urban	0.12	0.07	1.67	0.10	−0.02	0.27	1.13	0.98
Respondent occupation								
Not working	Base							
Working	0.36	0.08	4.34	0.00	0.20	0.52	1.43	1.22
Husband partner occupation								
Not working	Base							
Working	−0.09	0.30	−0.30	0.77	−0.69	0.51	0.91	0.50
Currently pregnant								
No	Base							
Yes	0.13	1.80E+13	0	1	−3.60E+14	3.60E+14	1.14	0.00
Duration of current pregnancy								
Second trimester	Base							
First trimester	0.10	0.23	0.42	0.67	−0.35	0.55	1.10	0.70
Third trimester	−0.20	0.24	−0.86	0.39	−0.66	0.26	0.82	0.51
Wealth index								
Poorest	Base							
Middle	0.27	0.10	2.60	0.01	0.07	0.47	1.31	1.07
Poor	0.09	0.10	0.94	0.35	−0.10	0.29	1.10	0.90
Richer	0.35	0.12	3.02	0.00	0.12	0.58	1.43	1.13
Richest	0.32	0.13	2.42	0.02	0.06	0.58	1.38	1.06
Current marital status								
Never in union	Base							
Divorce	0.20	0.36	0.57	0.57	−0.50	0.90	1.23	0.61
Living with a partner	0.59	0.35	1.70	0.09	−0.09	1.27	1.81	0.91
Married	0.47	0.35	1.37	0.17	−0.20	1.15	1.61	0.82
No longer living together/separated	0.59	0.34	1.73	0.08	−0.08	1.26	1.80	0.92
Widow	0.13	0.36	0.35	0.73	−0.58	0.83	1.13	0.56
Religion								
Others	Base							
Christianity	−0.09	0.16	−0.58	0.56	−0.40	0.22	0.91	0.67
Islam	0.01	0.17	0.06	0.95	−0.32	0.35	1.01	0.72
Traditionalist	0.13	0.23	0.54	0.59	−0.33	0.58	1.13	0.72
Completeness of current pregnancy								
Incompleteness of current pregnancy information	Base							
Completeness of current pregnancy information	0.13	1.80E+13	0	1	−3.60E+14	3.60E+14	1.14	0.00
Living children and current pregnancy								
5 or more living children + current pregnancy	Base							
1 Living child + current pregnancy	1.12	0.25	4.58	0.00	0.64	1.60	3.08	1.90
2 Living child + current pregnancy	0.81	0.30	2.71	0.01	0.22	1.40	2.25	1.25
3 Living child + current pregnancy	0.63	0.31	2.05	0.04	0.03	1.24	1.88	1.03
4 Living child + current pregnancy	0.00	0.26	0.01	0.99	−0.50	0.51	1.00	0.61
5 Living child + current pregnancy	0.89	0.08	11.17	0.00	0.74	1.05	2.44	2.09
Ethnicity								
Others	Base							
Akan	0.55	0.23	2.38	0.02	0.10	1.00	1.73	1.10
Ewe	0.80	0.24	3.37	0.00	0.34	1.27	2.24	1.40
Ga/Dangbe	0.59	0.25	2.35	0.02	0.10	1.08	1.80	1.10
Mole Dagbani	0.02	0.23	0.09	0.93	−0.42	0.46	1.02	0.65
Husband partner age								
15–24 years	Base							
25–34 years	−0.56	0.23	−2.39	0.02	−1.01	−0.10	0.57	0.36
35–44 years	−0.51	0.24	−2.14	0.03	−0.98	−0.04	0.60	0.37
45–54 years	−0.49	0.25	−1.98	0.05	−0.98	−0.01	0.61	0.37
55 years and above	−0.49	0.26	−1.87	0.06	−1.01	0.02	0.61	0.37
Number of living children								
0 living child	Base							
1 living child	−0.23	0.21	−1.14	0.26	−0.64	0.17	0.79	0.53
2 living child	−0.03	0.24	−0.11	0.91	−0.49	0.44	0.97	0.61
3 living child	−0.09	0.25	−0.37	0.71	−0.58	0.39	0.91	0.56
4 living child	0.43	0.18	2.38	0.02	0.08	0.78	1.53	1.08
5 living child	0.22	0.08	2.83	0.00	0.07	0.38	1.25	1.07

With husband partner education and termination of pregnancy had a higher association in men with secondary education (AOR = 0.83; 95% CI: 3.1) as compared with no education as the reference. The secondary education level is the one that is predicting the outcome variable with the highest OR.

## 6 DISCUSSION

Research question: What are the socio‐demographic factors on termination of pregnancy in Ghana.

This analysis assessed the socio‐demographic factors and other variables that predicts the termination of pregnancy in Ghana. This study identified age, type of place of residence, respondent occupation, currently pregnant, woman's individual sample weight, completeness of current pregnancy, living children + current pregnancy, ethnicity and number of living children are significant predictors of the outcome variable.

The analysis established that sociodemographic factors play a key role in the termination of pregnancy. Age had higher odds and is a strong predictor of the outcome variable. This agrees with the study by Adjei et al. (2015), who are of the opinion that sociodemographic factors, including age, marital status, employment status, wealth status, level of education, religious affiliation and place of residence, are frequently associated with the likelihood of having terminated a pregnancy.

Higher/Tertiary level of education. The analysis of the study established that, a level of education has a lower OR and an AOR, indicating that individuals with this level of education are less likely to have a certain outcome compared to the reference group of “No Education in the multivariable logistic regression”. This suggests that having a higher level of education is negatively associated with the outcome variable. In terms of the relationship between education and pregnancy termination, higher education may expose women to the risks associated with pregnancy termination and hence may reduce their involvement in them. On the contrary, a study in China by Geo et al. (2019), found pregnancy termination to be high among women with higher educational levels and argued that educated women may have pregnancies that interfere with their education and hence may decide to terminate those pregnancy. The other groups, “Primary” and “Secondary”, have OR >1, indicating that these groups are positively associated with the outcome variable.

With a husband partner education secondary education level had higher odds of terminating pregnancy, this indicates that women with husbands with secondary educational background were more likely to terminate their pregnancies compared to the reference category of no education. This confirms the works of Ahinkorah et al. (2021), who found out that the educational level or partner education influences termination of pregnancy.

The study found that the urban place of residence is the one that is predicting the outcome variable with the highest OR. This finding agrees with other works which state the likelihood of pregnancy termination was lower among young women who lived in rural areas as compared to those in urban areas (Adjei et al., 2015; Seidu et al., 2020).

The study has shown that “respondent currently working” predicts the outcome variable with the highest OR. In terms of wealth index, “Richer” and “Richest” levels of Wealth Index have a stronger association with the outcome variable. This suggests that these levels of Wealth Index may be good predictors of the outcome variable. With current marital status “Living with a partner” and “No longer living together/Separated” marital statuses have the largest positive effect on the outcome variable when compared to the reference category “Never in union”. This is to say that “respondent currently working”, Wealth Index and marital status are predictors of termination of pregnancy. This confirms the work of other researchers (Ahinkorah et al., 2021; Klutsey & Ankomah, 2014).

Using the multivariable logistic regression to analyse religion, the study found that in terms of religion practice, traditionalist religion are more likely to have a positive outcome, Christianity is less likely to have a positive outcome, for Islam is relatively close to zero, indicating that the effect of this religion on the outcome variable may be minimal. This agrees with a study in South Africa that reveals that religion play a role in the youths' attitudes towards abortion. While the Hindu subsample indicated higher overall support across the different scenarios, the Muslim subsample reported greater disapproval than the other groups on ‘Elective reasons’ and in instances of ‘Objection by significant others.’ The Christian youth had the most negative attitudes toward abortion for ‘Traumatic reasons’ and ‘When women's health/life’ was threatened (Selebalo‐Bereng & Patel, 2019). Individuals with five or more living children have higher odds of having a certain outcome compared to individuals with 0 living children, which is the reference group.

### 6.1 Limitations

The analysis has strengths and limitations; one of the strengths is the large sample size, which could be a representativeness of other women of reproductive age group in the general population, and thus making the result generalizable. The DHS dataset is standardized and validated, eliminating information bias. The limitation of using secondary source data is subject to missing important information (Variables) and recall bias.

## 7 CONCLUSION

The results indicated that age, type of place of residence, respondents' occupation, currently pregnant, woman's individual sample weight, completeness of current pregnancy, living children + current pregnancy, ethnicity and number of living children are significant predictors of termination of pregnancy. The study also confirms that improvement in education and eradicating poverty would be an important policy intervention to increase the reduction of pregnancy termination. The findings of the study also provide the needed information in designing health interventions in reducing termination of pregnancy. It is therefore highly recommended that the government and other health‐related agencies should focus more on reproductive health programmes targeted at those who are at risk of pregnancy termination as such programmes will contribute to attaining sustainable development goal 3, that seeks to reduce maternal mortality by 2030.

## AUTHOR CONTRIBUTIONS

M.A.S.A.‐M. contributed to the conception, design and drafting the manuscript and bears the primary responsibility for the content of the manuscript. I.A.‐M. contributed to data analysis. All the authors read and approved the content of the manuscript.

## FUNDING INFORMATION

There was no funding for this study.

## CONFLICT OF INTEREST STATEMENT

The authors declare that they have no competing/financial interests.

## ETHICS STATEMENT

Not applicable.

## CONSENT FOR PUBLICATION

Not applicable.

## Data Availability

The data that support the findings of this study are available from the corresponding author upon reasonable request.
